# Binding of a Pocket Factor to Hepatitis B Virus Capsids Changes the Rotamer Conformation of Phenylalanine 97

**DOI:** 10.3390/v13112115

**Published:** 2021-10-20

**Authors:** Cihan Makbul, Christian Kraft, Matthias Grießmann, Tim Rasmussen, Kilian Katzenberger, Melina Lappe, Paul Pfarr, Cato Stoffer, Mara Stöhr, Anna-Maria Wandinger, Bettina Böttcher

**Affiliations:** 1 Rudolf Virchow Center, Center for Integrative and Translational Bioimaging, University of Würzburg, 97080 Würzburg, Germany; cihan.makbul@uni-wuerzburg.de (C.M.); christian.kraft@virchow.uni-wuerzburg.de (C.K.); matthias.griessmann@uni-wuerzburg.de (M.G.); tim.rasmussen@uni-wuerzburg.de (T.R.); 2 Biocenter, University of Würzburg, 97074 Würzburg, Germany; kilian.katzenberger@stud-mail.uni-wuerzburg.de (K.K.); melina.lappe@stud-mail.uni-wuerzburg.de (M.L.); paul.pfarr@stud-mail.uni-wuerzburg.de (P.P.); cato.stoffer@stud-mail.uni-wuerzburg.de (C.S.); mara.stoehr@stud-mail.uni-wuerzburg.de (M.S.); anna-maria.wandinger@stud-mail.uni-wuerzburg.de (A.-M.W.)

**Keywords:** Hepatitis B Virus, pocket factor, Triton X 100, envelopment, maturation signal, single strand blocking, electron cryo-microscopy, isothermal titration calorimetry

## Abstract

(1) Background: During maturation of the Hepatitis B virus, a viral polymerase inside the capsid transcribes a pre-genomic RNA into a partly double stranded DNA-genome. This is followed by envelopment with surface proteins inserted into a membrane. Envelopment is hypothetically regulated by a structural signal that reports the maturation state of the genome. NMR data suggest that such a signal can be mimicked by the binding of the detergent Triton X 100 to hydrophobic pockets in the capsid spikes. (2) Methods: We have used electron cryo-microscopy and image processing to elucidate the structural changes that are concomitant with the binding of Triton X 100. (3) Results: Our maps show that Triton X 100 binds with its hydrophobic head group inside the pocket. The hydrophilic tail delineates the outside of the spike and is coordinated via Lys-96. The binding of Triton X 100 changes the rotamer conformation of Phe-97 in helix 4, which enables a π-stacking interaction with Trp-62 in helix 3. Similar changes occur in mutants with low secretion phenotypes (P5T and L60V) and in a mutant with a pre-mature secretion phenotype (F97L). (4) Conclusion: Binding of Triton X 100 is unlikely to mimic structural maturation because mutants with different secretion phenotypes show similar structural responses.

## 1. Introduction

Hepadnaviridae are ancient, enveloped DNA-viruses that infect most bony vertebrates [1]. One member of this family is the Human Hepatitis B virus (HBV), which causes chronic and acute liver disease. HBV is circulating in humans at least since Neolithic ages more than 7000 years ago [2]. In modern history, more than a third of the world’s population has been infected with HBV [3] before the start of the world-wide vaccination programs.

Mature HBV consists of a membranous envelope, which is densely packed with three types of surface proteins (HBs). These surface proteins are referred to as small (S-HBs) medium (M-HBs) and large (L-HBs) and have a molar ratio of approximately 4:1:1 within the viral envelope [4]. S-HBs forms the common C-terminal core of the three HBs types and consists of four transmembrane helices. M-HBs and L-HBs have *N*-terminal extensions of increasing length. The envelope with the surface proteins surrounds an icosahedral capsid that is formed by 240 copies of the core protein (HBc). This capsid encloses the viral polymerase (protein P) together with the partly double-stranded, relaxed and circular DNA-genome.

During viral maturation, protein P transcribes the genome from a pre-genomic RNA precursor (pgRNA) inside the viral capsid. Capsids with a mature genome are enveloped and secreted together with an excess of sub-viral particles [5] formed by HBs. Early investigations on Duck Hepatitis B Virus (DHBV) showed that secreted capsids contain exclusively mature, partly double stranded DNA genomes whereas capsids in the host cell harbor genomes in all maturation states [6,7]. This led to the assumption that a maturation signal renders capsids competent for envelopment after full maturation of the genome [7]. Later, it was shown that not only capsids with a mature genome are enveloped and secreted but also empty capsids [8], which account for the majority of all secreted capsids. This observation led to the single strand blocking hypothesis, suggesting that capsids with an immature genome display a blocking signal that prevents envelopment [9]. It is plausible that such determinants for a maturation or blocking signal are encoded in the structure of the capsids. Even though many structures of capsids have been determined [10,11,12,13,14,15,16,17,18,19], the nature of the blocking signal remained enigmatic.

The building block of the capsids are dimers of HBc, which consist of an N-terminal alpha-helical assembly domain and a largely unstructured C-terminal domain (CTD). The CTD is rich in positively charged Arginines and, therefore, is often referred to as an Arginine Rich Domain (ARD). The ARD is important for maintaining the electrostatic balance with the negatively charged nucleic acids and is essential for the complete encapsidation of the pgRNA [20,21]. Six phosphorylation sites are interspersed in the ARD. Phosphorylation of these sites counterbalances the positive charges of the ARD and abolishes RNA-packing, which eventually leads to empty capsids [8,12,22]. In capsids containing pgRNA, de-phosphorylation and encapsidation occur concomitantly [23].

While the ARD is projecting towards the capsid interior, the NTD forms the capsid shell with protruding dimeric spikes that contact the membranous envelope [24,25]. These spikes consist of a four-helix bundle, to which each subunit contributes two helices. The intra dimer contact is further stabilized by an SS-bridge between the two opposite Cys-61 in the center of the spike. This SS-bridge forms rapidly in capsid like particles (CLPs) [26] and separates the protruding part of the spike from the part, which is embedded in the continuous capsid shell. The protruding part is structurally highly dynamic [27,28] and adapts its conformation to binding of inhibitory peptides [10,29] or fragments of HBs [30] by splaying the spike helices apart. In contrast, the embedded part of the spike is structurally rigid and shows little response to conformational challenges at the tips of the spikes.

Although in contact with the envelope, mutations at the tips of the spikes have little impact on envelopment or secretion of HBV [31]. Instead, most of the mutations with a secretion phenotype map to a hydrophobic pocket in the center of the spikes below the SS-bridge in the rigid part of the assembly domain. Naturally occurring point mutations like HBc-F/I97L with an immature secretion phenotype [32,33] or point mutations with a low secretion phenotype, such as HBc-L60V and HBc-P5T [34], are located in the area surrounding the pocket. Regardless of the differences in phenotype, the structural changes between these mutants are restricted to the respective residue without long-range impact on the protein backbone [10,13] offering no plausible hint of the possible mode of action of maturation signals or blocking signals.

New evidence for the existence of two alternate conformations that could provide such maturation or blocking signals comes from solid state NMR-studies [35]. These alternate conformations can be switched by binding a pocket factor to the hydrophobic pocket in the center of the spikes [35]. The pocket factor can be mimicked by Triton X 100 (TX100), which is a detergent commonly used in cell lysis during purification of HBc-CLPs. Lecoq and coworkers showed that the point mutations L60W and P5W, which block the access to the pocket, support the maturation of the genome but not the progression into enveloped virus particles [35]. We were intrigued by this simple concept of adding TX100 for switching CLPs into a supposedly envelopment competent state. We asked what the underlying conformational changes were and whether these changes differed between CLPs of wt HBc, mutants with immature secretion phenotype (HBc-F97L) or mutants with low secretion phenotypes (HBc-P5Tand HBc-L60V). To address this question, we used electron cryo-microscopy and image processing and compared HBc-CLPs from detergent-free purifications [10] with those purified in the same way with added TX100 after the purification process.

## 2. Materials and Methods

### 2.1. Purification of HBc-CLPs 

The purification of HBc-CLPs was previously described [10]: In brief, wt HBc and HBc-F97L, HBc-P5T and HBc-L60V were overexpressed in BL21 (DE3) Star cells. After harvesting by centrifugation, the cells were disrupted by microfluidization (M-110P Microfluidizer, Westwood, MA, USA) at 1500 bar. The cell lysate was cleared by centrifugation and the supernatant was treated by two subsequent ammonium sulfate precipitations to enrich CLPs. The ammonium sulfate precipitates were dissolved in buffer und further purified by a sucrose density step-gradient centrifugation at 125,000× *g* for 4 h. Fractions from the density step-gradient were confirmed for the presence of intact and pure CLPs by SDS-PAGE, native agarose gel electrophoresis (NAGE) and electron microscopy of stained samples. CLPs were stored at 4 °C. No detergents were used during purification.

### 2.2. Isothermal Titration Calorimetry (ITC)

A 1.2 L buffer A (0.2 M Na_2_HPO_4_, 50 mM NaCl, pH 7.5) was filtered (220 nm pore size filter, Rotilabo syringe filter, Carl Roth GmbH + Co. KG, Karlsruhe, Germany) and was degassed for 20 min. Then, 3 mL of purified HBc CLPs from the sucrose density gradient was transferred into a membrane tubing with an MWCO of 1 MDa (Spectra/Por Biotech cellulose ester tubing, Spectrum Laboratories Inc., Rancho Dominguez, CA, USA) and dialyzed against buffer A under gentle stirring at 4 °C for 16 h. The dialyzed sample was concentrated (molecular weight cutoff (MWCO) 30 kDa, Spin-X UF 6 mL, Corning Inc., Corning, NY, USA) at 4 °C and the protein concentration was determined by the Bradford assay (Roti ^®^ Nanoquant, Carl Roth GmbH + Co. KG, Karlsruhe, Germany).

The concentrated samples were degassed again for 10 min at 20 °C (ThermoVac, Malvern Panalytical, Malvern, Worcestershire, UK) before starting the ITC experiment. Four independent ITC experiments were performed at 37 °C with a VP-ITC instrument (Microcal Inc., Northampton, MA, USA). The protein concentration of HBc in the ITC cell was 60 µM (monomer concentration) for all four experiments. The concentration of TX100 titrated into the ITC cell was varied between 400 µM and 600 µM. The ITC isotherms were fitted with a single binding-site model by NITPIC/SEDPHAT and were plotted with GUSSI [36]. The thermodynamic parameters of all four experiments were averaged and their standard deviation was calculated.

### 2.3. Sample Preparation for Electron Cryo-Microscopy

For electron cryo-microscopy, 200 µL from a density gradient fraction with verified CLPs was diluted 5-fold with buffer B (40 mM HEPES, 200 mM NaCl, 1 mM MgCl_2_, 1 mM CaCl_2_, pH 7.5) and concentrated at 4000× *g* and 4 °C with 0.5 mL centrifugal filters (Amicon™ Ultra, Merck KGaA, Darmstadt, Germany) with a MWCO of 100 kDa. Fresh buffer B was added to the concentrate, thoroughly mixed and concentrated again. This procedure was repeated 2–3 times until the sucrose concentration dropped below 0.3%. The protein samples were centrifuged once by centrifugal filter units (Ultrafree MC, 100 nm pore size, VV 0.1-m, Merck KGaA, Darmstadt, Germany) and their concentrations were determined by the Bradford assay (Roti ^®^ Nanoquant, Carl Roth GmbH + Co. KG, Karlsruhe, Germany). 10 mM TX100 (Sigma Aldrich Chemie GmbH, Steinheim, Germany) was dissolved in buffer B and mixed with HBc samples. For wt HBc, the final concentrations were 170 µM HBc-monomer and 305 µM TX100. For all other variants, the final concentration of TX100 was 2 mM and the HBc-monomer concentrations were 700 µM for HBc-P5T and HBc-F97L, and 400 µM for HBc-L60V, which is less soluble.

Copper grids coated with holey carbon film (300 mesh, R 1.2/1.3., Quantifoil Micro Tools, Jena, Germany) were treated with a plasma cleaner (Harrick Plasma, model PDC-002, Ithaca, NY, USA) at an air pressure of 29 Pa for 2 min at medium-power. 3.5 µL of protein sample was applied onto the grids and vitrified in liquid ethane using a Vitrobot mark IV (FEI Company, Hillsboro, OR, USA). The same settings of the Vitrobot were used for all samples (blot force of 25, wait and drain times of 0 s, blot time of 6 s, nominal humidity of 100% at 4 °C, blotting filter paper Whatman 541). After the vitrification all samples were stored in liquid nitrogen for at least 12 h before loading into the electron microscope.

### 2.4. Electron Cryo-Microscopy

Movies of vitrified HBc-CLPs were acquired semi-automatically with the software EPU on a Krios G3 electron microscope equipped with a Falcon III camera (Thermo Fisher Scientific, Hillsboro, OR, USA) as described before [10,37] with the following modifications. For all experiments, the total exposure was 40 e^−^/Å². The exposure of a movie was fractionated across 20 fractions. At one stage position, 15 movies were acquired using image shifts. The acquisition area covered the central hole and the four closest neighboring holes. Three movies were recorded per hole. Once per mesh it was waited that specimen drift dropped below 2 Å/s. Autofocusing was carried out after stage movements of more than 3–5 µm. This gave a throughput of approximately 265 micrographs per hour similar as described for the aberration free image shift (AFIS) strategy [38] but without compensating for the image shift induced beam tilt.

### 2.5. Image Processing

Fractions of movies were exposure weighted, motion corrected and averaged with motioncor2 [39]. The defocus of the averaged movies was determined with ctffind4 [40]. Movie averages together with ctf-parameters from ctffind4 were imported into relion 3.1 [41] and processed in 15 optic groups. Each image shift position was assigned to a separate optic group to enable image shift specific beam tilt corrections. Subsequent image processing was carried out with relion 3.1 [41] as described before [10]. CTF-refinement (“relion_ctf_refine”) of beam tilt and per particle defocus followed by “relion_refine” improved the resolution. An additional round of “relion_ctf_refine” followed by “relion_refine” showed only marginal improvements and was omitted in some of the datasets.

The overall resolution was estimated by Fourier-Shell Correlation between masked half-maps at the end of gold standard refinement at a correlation cut-off of 0.143 using “relion_postprocess” [42]. In case that the estimated resolution was better than 2.9 Å, maps were re-reconstructed correcting for the curvature of the Ewald’s-sphere with “relion_reconstruct” [43,44], which led to slight improvements in resolution and in the map density.

Final maps were sharpened with “relion_postprocess” using the previously determined modulation transfer function of the Falcon III camera [37] and the estimated B-factors.

For modelling and illustration purposes, small boxes with a box-size of 128^3^ voxel (“small map”) were excised from the post-processed maps centered at one asymmetric unit using “relion_image_handler”. These maps covered only part of the capsid shell. To minimize the differences between maps from reconstructions of different HBc samples, the “small maps” were scaled in their amplitude profile to a common reference map using the option “adjust_power” of “relion_image_handler”. The reference map was the “small map” of wt HBc low-pass filtered to 3.2 Å resolution. These “power-adjusted maps” were used for calculating difference maps and local correlation maps with Chimera. Figures of maps and models were generated with Chimera [45] or ChimeraX [46].

### 2.6. Modelling of Cryo-EM Maps and their Validation

These “small maps” were density modified to maximize the clarity of the maps by “Phenix Autosharpen Map” [47]. For the modelling of HBc with bound TX100 or with bound TX100 and bound peptide “SLLGRM” the PDB-model 7OD4 [10] was fitted as a rigid body into the density-modified maps and modified by Coot [48]. The geometrical restraints library of the TX100 fragment (pdb code: TRT) from the Grade Web Server (grade.globalphasing.org) were loaded into Coot, which was used for modelling and refinement. Only regions of density modified maps above 1σ were modelled. Finally, the resulting models were real space refined and validated with Phenix [49] using the geometrical restraints library of the TX100 fragment.

## 3. Results

**Figure 1:**
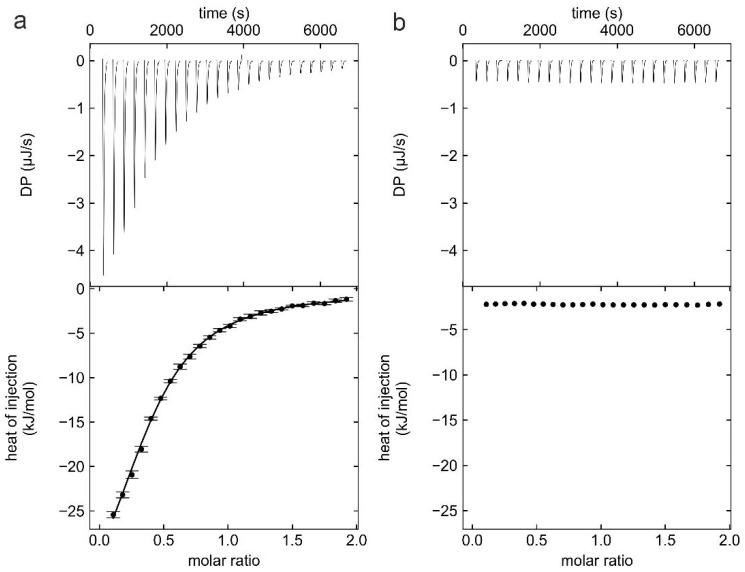
Thermodynamic characterization of binding of TX100 to HBc-CLPs by ITC. (**a**) A typical isotherm of titration (upper panel) with 600 µM of TX100 into a solution of 60 µM HBc (monomer concentration). The ITC isotherms were fitted with a single binding-site model by NITPIC/SEDPHAT and plotted by GUSSI [36] (lower panel). (**b**) Isotherm of dilution of 600 µM TX100 into dialysis buffer in the ITC cell to evaluate the heat contribution of TX100 dilution. The final concentration of TX100 at the end of the experiment in the ITC cell was 96 µM, which is below the CMC of TX100 (CMC = 220 µM, determined by ITC, not shown).

Binding of wt HBc-CLPs to TX100 was quantified by Isothermal Titration Calorimetry (ITC) (Figure 1). TX100 bound to wt HBc-CLPs with a dissociation constant of K_D_ = 11.6 ± 0.4 µM. The binding was exothermic with ∆H = 29 ± 10 kcal/mol without relevant, entropic contribution to the binding (∆S = 0 ± 30 cal/(mol K)).

The K_D_ value of the TX100 wt HBc-CLP interaction at 310 K was similar to the K_D_ of 8.3 ± 0.5 μM previously reported for the interaction of TX100 with reassembled C-terminally truncated HBc_1–149_ CLPs at 298 K [35]. This suggested that binding determinants for TX100 were independent of whether CLPs had packaged RNA (wt HBc), were lacking the ARD (HBc_1–149_) or originated from cell based or in vitro assembly. This is in line with the previous reports from solid-state NMR investigations, which showed little differences in the chemical shift deviations between these species [35].

**Figure 2:**
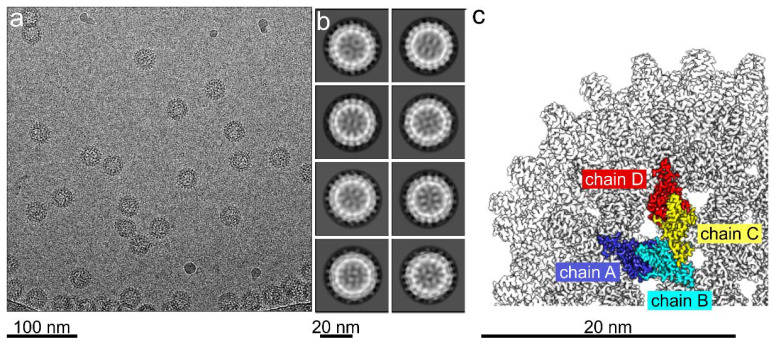
Electron cryo-microscopy of 170 µM wt HBc-CLPs with 300 µM TX100. (**a**) Micrograph of vitrified HBc-CLPs with added TX100. (**b**) 2D-class averages of the 8 most populated classes. (**c**) Close-up of the surface representation of the 3D-map of HBc-CLPs with added TX100. The map is filtered to a nominal resolution of 3.1 Å and B-factor sharpened by 100 Å². One asymmetric unit is colored and highlights the four monomers in spatially different surroundings. The monomers in the different surroundings are referred to as chain A (blue), chain B (cyan), chain C (yellow) and chain D (red). Scale bars are shown below.

**Figure 3:**
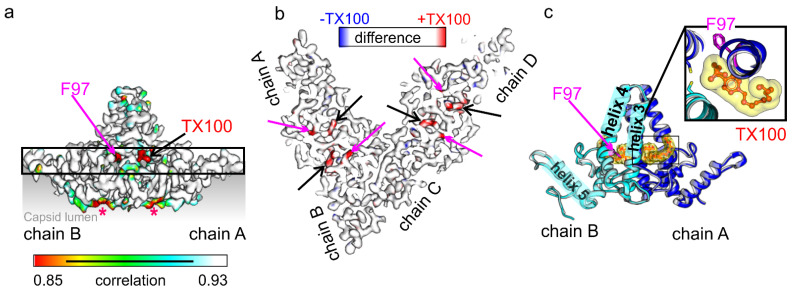
HBc with added TX100 (a) Surface representation of the map masked around the AB-dimer. The map is colored with its local correlation with the map without TX100 (EMD12819, [10]). For calculating the local correlation, “power-adjusted maps” were used. The “power-adjusted maps” were also locally aligned around the presented AB-dimer to compensate for subtle variations in the diameter of the CLPs. The color key indicates the local correlation. Areas of low local correlation map to Phe-97 (magenta arrow and label) and TX100 (black arrow, red label). Other areas of low local correlation (red stars) are located in the capsid lumen, which is shaded in grey. This region is likely to account for modifications by glutathione or binding of the C-terminal Cys-183 to either Cys-107 or Cys-48 as previously reported [13]. Differences do not correlate with the presence or absence of TX100 (not shown). The black scale bar inside the color key indicates 5 nm. (**b**) Slab through the asymmetric unit (chains A-D) viewed towards the capsid interior. The position and thickness of the slab is marked in (**a**) by a black square. The slab is 1 nm thick and colored with the differences between maps of HBc with added TX100 (red if larger density, see color key above) and without TX100 (blue if larger density). Black arrows mark the position of additional density that we attribute to TX100 inside the pockets. The red arrows mark the new side chain position of Phe-97. (**c**) Model of the AB-dimer shown as cartoon representation with TX100 in yellow in surface representation. The position of Phe-97 is indicated by a magenta arrow. Helices 3–5 are labelled in chain B (cyan) and follow the previous assignment in [16]. The close-up shows a view along the dimer axis centered on the TX100 molecule in chain A. The approximate position of the close-up is indicated as a black square in the dimer.

Next, we determined the structure of HBc-CLPs with bound TX100 by electron cryo- microscopy. Electron micrographs (Figure 2a) showed similar CLPs of HBc with added TX100 as previously observed for CLPs purified in the same way but without TX100 [10]. One of the marked differences was the lower particle density of TX100 containing samples compared to detergent-free samples at a similar protein concentration. We attribute this difference to a reduced interaction with the air water interface in the presence of detergent as previously described for other systems [50,51]. The 2D-class averages showed that most CLPs had dimensions consistent with capsid formed by 240 subunits in a T = 4 assembly (Figure 2b). We calculated 3D-maps of these CLPs to a resolution of 3.1 Å (Table 1), which showed the expected two dimers per asymmetric unit (Figure 2c). Differences to the previously determined map of HBc CLPs in the absence of TX100 (EMD12819, [10]) accumulated around the hydrophobic pockets, which contained additional densities (Figure 3).

**Figure 4:**
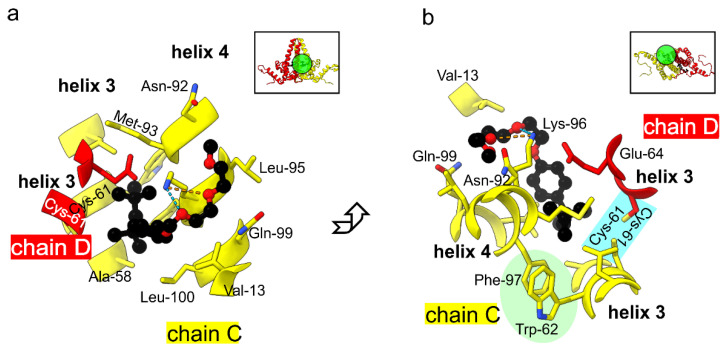
One of the hydrophobic pockets with bound TX100 in the CD-dimer. (**a**,**b**) show two views, which are perpendicular to each other. The general viewing direction of the pocket is shown in the inserts in the upper left corner of the panels. The approximate positions of the close-ups below are indicated with circles filled in green. The close-ups show TX100 in black in ball and stick representation together with all residues within a distance of 4 Å. TX100 is located at the dimer interface between chains D (red) and chains C (yellow). The head group of TX100 is close to Cys-61 of both chains at the dimer interface (marked in blue in (**b**)). Phe-97 is in an orientation where it supports π-stacking with Trp-62 ((**b**), marked in green). Lys-96 makes H-bonds with the oxygens in TX100 (dashed line).

To evaluate the structural changes in TX100 binding, we refined the model of the TX100 free HBc (7od4, [10]) into the map of HBc with bound TX100. This showed that the protein backbone was unaffected by the binding of TX100 as expected from the high local correlation between the maps with and without added TX100 (Figure 3a). The densities in the hydrophobic pockets were modelled as TX100 with the hydrophobic 4-(1,1,3,3-tetramethylbutyl)-phenyl group inside the pocket at the dimer interface. The closest side chain residues within 4 Å of the hydrophobic head group were Leu-60, Cys-61 and Glu-64 in helix 3 of one subunit. The other subunit contributed Ala-58, Cys-61 Trp-62, Leu-65 in helix 3, Asn-92, Met-93, Lys-96, Phe-97, Arg-98, Leu-100 in helix 4 and Pro-5, Tyr-6 at the N-terminus. The hydrophilic part of TX100 is a poly-ethylene-glycol with 9-10 ethylene oxide repeats. Only the first one to two repeats were accommodated by the difference density of WT-HBc +/− TX100 while the remaining repeats remained unresolved. The resolved ethylene oxide repeats were positioned between the side chains of Gln-99 and Lys-96 in helix 4 at the outside of the spike. Lys-96 formed H-bonds with the oxygens in the first ethylene oxide repeats (Figure 4).

**Figure 5:**
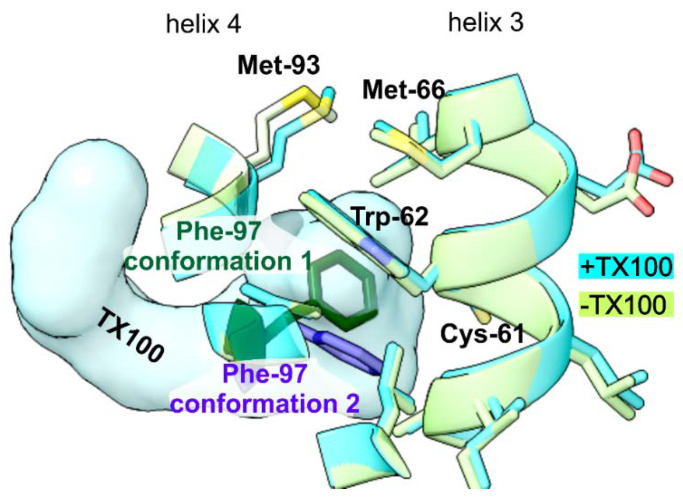
Close-up of models of HBc-CLPs with added TX100 (+TX100,cyan) and HBc-CLPs without TX100 (-TX100, green, 7OD4, [10]) superimposed on top of each other. The close-up is centered on Trp-62 in chain B and TX100 (shown in space fill representation). Both models differ only in the rotamer conformation of Phe-97. In the absence of TX100, Phe-97 points towards Cys-61 (green, “conformation 1”). In the presence of TX100, TX100 occupies the position of Phe-97 side chain in “conformation 1” and, Phe-97 (blue, “conformation 2”) makes a π-stacking interaction with Trp-62, which is further extended by Met-66 towards the tip of the spike.

These residues in the immediate vicinity of TX100 were also identified in the previous study by solid state NMR as having large chemical shift deviations between the two conformational sates [35]. To differentiate whether these residues changed their conformation in addition to interacting with TX100, we compared the models with and without bound TX100 guided by the differences and low local correlation between the maps of HBc +/− TX100. The only residue with a noticeable difference was Phe-97, which had changed its rotamer conformation (Figure 5). In the absence of TX100, Phe-97 pointed towards the gap between the two Cys-61 at the dimer interface in the center of the spike (“conformation 1”). In the presence of TX100, this position was occupied by the hydrophobic 1,1, di-methyl group of TX100 and Phe-97 adopted a different rotamer conformation pointing towards the side of the spike (“conformation 2”). In this conformation Phe-97 made a π-stacking contact with Trp-62 on helix 3. This stack was further extended by Met-66 on helix 3.

**Figure 6:**
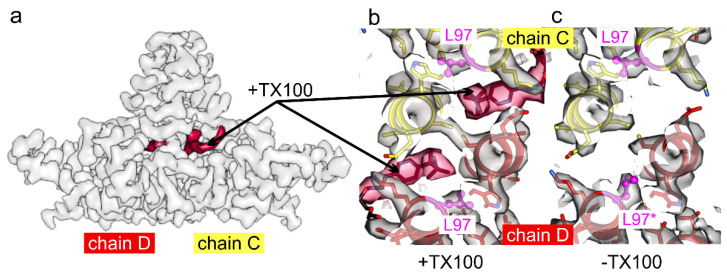
HBc-F97L with and without added TX100. (**a**) Surface representation of the map of HBc-F97L with added TX100: The map covers the CD-dimer, the rest is omitted for clarity. The density in the hydrophobic pocket is attributed to TX100 and is colored in red. (**b**,**c**) slab of the maps and models of F97L-HBc through the hydrophobic pocket with added TX100 (**b**) and without TX100 (**c**). Chain D is shown in red and chain C in yellow. Leu-97 is shown in magenta in ball and stick representation. Without TX100, Leu-97 in chain D (L97*) is in a conformation akin to “conformation 1” of Phe-97 in wt HBc. All other Leu-97 in (**b**,**c**) are in a conformation similar to “conformation 2” of Phe-97 in wt HBc. The surface representations show the “power-adjusted maps” calculated at the same density threshold.

Intrigued by the changes in the rotamer conformation of Phe-97, we revisited the mutant HBc-F97L, which is a naturally occurring variant with an immature secretion phenotype [32,33]. In an earlier study [13] the side chain of Leu-97 was in the same position as Phe-97 in “conformation 2” of the TX100 bound state of wt HBc-CLPs. However, this earlier study also identified a pocket factor with an L-shaped EM-density similar to the hydrophobic head group of TX100. Thus, we suspected that this earlier map represents HBc-F97L in a TX100 bound state, which is plausible as TX100 was used during cell lysis. In contrast, the map of HBc-F97L (EMDB 4417 [37] and all maps generated in this study (Table 1), (Figure 6) were reconstructed from HBc-CLP purifications without TX100 (and any other detergents) and did not show a pocket factor. Interestingly, in the absence of TX100, the side chain densities of Leu-97 in chains A and D (Figure 6c, L97*) pointed towards the center of the spike similar as Phe-97 in “conformation 1” of wt HBc whereas the densities of Leu-97 in chains B and C pointed towards the side of the spike similar as Phe-97 in “conformation 2” of wt HBc (Figure 4). Thus, the two Leu-97 in a dimer are in different rotamer conformations in the absence of TX100.

We added TX100 to the detergent-free purified HBc-F97L CLPs followed by electron cryo-microscopy and image processing. The map at 2.9 Å resolution (Table 1) showed density in all hydrophobic pockets with the characteristic L-shape of TX100. All side chains of Leu-97 were in the alternate rotamer conformation akin to “conformation 2” (Figure 6b).

**Figure 7:**
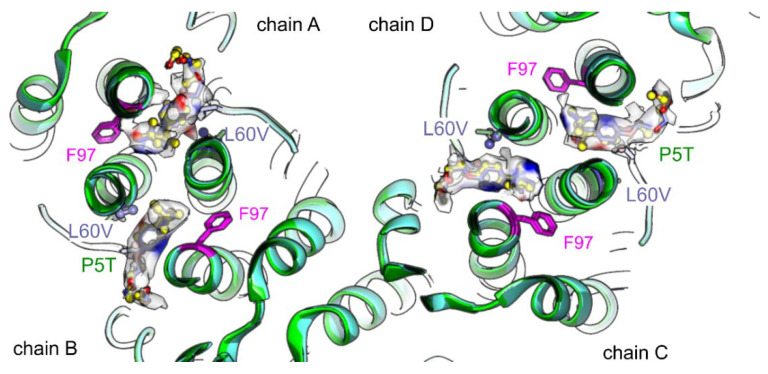
Superposition of models of HBc-L60V (blue) and HBc-P5T (green) with added TX100. The view shows a close-up of the asymmetric unit with chains A, B, C and D and is centered at the hydrophobic pocket. The side chains of Phe-97 (magenta), Pro/Thr-5 and Leu/Val-60 (dark-blue for HBc-L60V and dark green for HBc-P5T) are shown for both models. TX100 (dark blue in HBc-L60V and yellow in HBc-P5T) is shown together with its surrounding map densities. The densities are colored with the differences between both maps (red: additional density in HBc-L60V and blue additional density in HBc-P5T). For difference imaging both maps were locally aligned.

Next, we looked at the mutants HBc-P5T and HBc-L60V, which have a low secretion phenotype. Previous studies have shown that CLPs of both mutants are structurally almost indistinguishable from wt HBc [10] with the exception that Phe-97 adopts both alternate rotamer conformations with similar occupancy in HBc-L60V. The maps of both mutants with added TX100 showed TX100 in the hydrophobic pockets at the same positions as in wt HBc and in HBc-F97L (Figure 7). Phe-97 in both mutants was in “conformation 2”.

**Figure 8:**
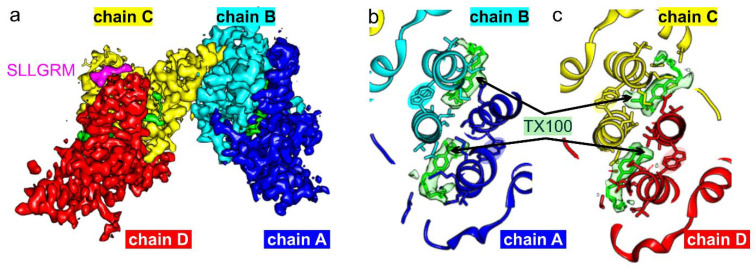
Wt HBc-CLPs with bound TX100 and peptide “SLLGRM”. (**a**) Surface representation of the asymmetric unit of HBc-CLPs. The density attributed to “SLLGRM” is shown in magenta and the density attributed to TX100 in green. Coloring of the chains is the same as in Figure 2. (**b**,**c**) close-up of the model at the hydrophobic pocket. TX100 is surrounded by its attributed map density (light green). The side chains within 4 Å of the TX100 molecules are shown. The AB-dimer (**b**) and the CD-dimer (**c**) are aligned to show the dimers in the same orientation. The π-stack of Phe-97 with Trp-62 is highlighted with background color similar to the respective chains.

Finally, we wondered whether the binding of TX100 affected binding of peptides at the tips of the spikes of the CLPs. To test this, we chose a minimal peptide binder “SLLGRM”, which binds only to the CD-spikes but not to the AB-spikes in CLPs of wt HBc [10]. In the absence of TX100, binding of “SLLGRM” to HBc-CLPs mobilizes Phe-97 in chain D, which adopts both possible conformations. This suggests that there is some cross-talk between binding in the hydrophobic pocket and the tips of the spikes. To establish whether TX100 affects the binding of “SLLGRM” by forcing Phe-97 into conformation 2, we added an excess of TX100 together with “SLLGRM” to wt HBc-CLPs before vitrification and imaging (Table 1). The map showed that all pockets were occupied by TX100 and that all Phe-97 had changed their conformation into “conformation 2” (Figure 8). “SLLGRM” bound to the tips of the CD-spikes but not to those of the AB-spikes. Thus, forcing Phe-97 into “conformation 2” was not sufficient to enable the binding of “SLLGRM” to the AB-spikes or abolish binding of “SLLGRM” to the CD-spikes.

## 4. Discussion

We found that TX100 binds to the hydrophobic pockets irrespective of the secretion phenotype of the HBc variant. The 1,1 di-methyl-group of TX100 sterically clashes with the side chain of residue 97 in HBc and forces it into the alternate “conformation 2”. This conformation is favorable for a π-stacking interaction between Phe-97 and Trp-62 and is further extended with Met-66. Such a stacking of aromatic side chains with Methionine is a frequent structural motif [53] for long range structural coupling. In the case of HBc-CLPs, the stacking in the TX100 bound state couples Phe-97 in the rigid domain of helix 4 with helix 3 at the mobile tip of the spike. This mobile part is delineated by residues Gly-63 in helix 3 and Gly-94 in helix 4 and changes its conformation upon binding of certain peptides [10,29] or in some mutants such as Y132A-HBc [54] or D78S-HBc [55]. The conformational changes of the mobile part lead to the splaying of the helices at the tips of the spikes generating a shallow groove at the dimer interface. Thus, the potential structural coupling between helix 3 and helix 4 in “conformation 2” provides an attractive mode of action how a pocket factor could change binding properties at the remote tips of the spikes. However, neither the mutants (P5T and L60V) nor wt HBc-CLPs with bound peptides show Phe-97 is in “conformation 2” suggesting that flipping of Phe-97 is dispensable for conformational changes at the tips of the spikes. Furthermore, forcing Phe-97 into “conformation 2” by binding of TX100 has no noticeable effect on binding of the minimal peptide binder “SLLGRM”. Thus, the binding sites at the tips of the spikes and in the hydrophobic pocket have no obvious conformational cross-talk and can be targeted independently or simultaneously. However, as the HBc-dimer is known to be highly allosteric, the binding of the detergent may activate a dynamical communication pathway between the rigid domain and the spike, which may be relevant to other capsid functions.

While NMR data [35] suggest that there is an extended conformational change between the pocket factor inducible conformational state A and the other state B, we saw only localized differences in the rotamer conformation of Phe-97 between “conformation 1” and “conformation 2”. Other than presented here, the previous NMR study used substantial amounts of TX100 during cell lysis probably generating a mixed population of CLPs with and without bound TX100. Consequently, the pocket factor free state B was enriched by procedures that deplete TX100 such as size exclusion chromatography or reassembly of capsids from dimers. Therefore, we suspect that the large changes between state A and B represent mainly the presence or absence of TX100. This is further supported by our EM-maps that show TX100 in the immediate vicinity of most residues that change their conformation according to the NMR measurements.

TX100 binds to a very rigid region of HBc-CLPs with low B-factors. Essentially, the interaction mechanism of TX100 with HBc-CLPs resembles binding of a ligand to a rigid platform inducing only slight alterations of surrounding rotamers without inducing medium or long-range conformational changes of the protein backbone. In contrast, binding of inhibitory peptides [10] induces large conformational changes in the protein backbone at the mobile tips of the spikes. Thus, the nature of the binding platforms at the tips and in the center of the spikes are very different.

Although the conformational changes of side chains induced by the pocket factor appear to be more localized than anticipated by the previous study [35], it is still interesting to note that small molecules such as TX100 bind with higher affinities to the hydrophobic pocket than peptide binders to the tips of the spikes [10,56]. Hence, the hydrophobic pocket provides a potent binding site that could interact with cellular factors. We have determined the druggability score of the pocket with DoGSiteScorer [57]. DoGSiteScorer assigned both TX100 binding sites within a dimer to a single pocket with a score of 0.8. This means that the pocket is highly druggable. A similar, albeit somewhat lower score in HBc is only found for the inter-dimeric pocket, which binds capsid assembly modulators [58,59]. Whether addressing the druggable TX100-binding site has a therapeutic benefit remains to be determined.

To identify which naturally occurring molecule could be mimicked by TX100, we searched for crystal structures with bound TX100 and came across the undecaprenyl pyrophosphate synthase, which has two TX100 molecules instead of prenyl-groups in its active site. One of the TX100 molecules is in an orientation where its hydrophobic head group is in contact with several Phenylalanines [60] similar as Phe-97 in HBc. Likewise, in the prenyl-binding protein, a Phenylalanine controls the size of the prenyl-binding pocket by flipping its side chain orientation upon substrate binding [61]. Thus, the hydrophobic pocket in HBc has some properties that are reminiscent of proteins that bind prenyl-groups, which are generally implicated in membrane trafficking. So far, prenylation is not reported for HBV proteins. However, it occurs for the Hepatitis D Virus, which depends on the prenylation of its large antigen for envelopment by HBs of HBV (reviewed in: [62]).

It is hard to reconcile how a pocket factor that is mimicked by TX100 could act as maturation signal considering that we do not see any major differences in the structural mode of binding in naturally occurring mutants (F97L, P5T and L60V) with different secretion phenotypes. It also appears questionable whether a maturation or blocking signal exists at all. Secreted HBV particles have been observed with their genome in all maturation states [63] albeit with pgRNA or immature genomes being several magnitudes rarer than those with a mature genome. In the absence of a blocking or maturation signal such differences in abundance could be explained simply if transcription of the RNA into DNA would be faster than trafficking of the capsids to the endo-plasmatic reticulum followed by envelopment. Thus, rather than postulating genome maturation dependent conformational changes in the capsid, secretion phenotypes might simply be explained by the speed of trafficking and envelopment.

## Figures and Tables

**Table 1:** Summary of image analysis.

Sample	# of Movies ^(3)^	# of Selected Particles	# of Particles in Final Map	Resolution of Map ^(1)^	B-Factor ^(2)^
HBc + TX100	6750	69,257	41,478	3.1 Å	121 Å²
HBc +TX100 + SLLGRM	1520	19,232	11,525	3.2 Å	102 Å²
HBc-F97L + TX100	5696	146,930	89,978	2.9 Å	121 Å²
HBc-F97L	2409	180,259	114,274	2.8 Å	107 Å²
HBc-L60V +TX100	6675	108,843	73,540	2.8 Å	106 Å²
HBc-P5T + TX100	5625	16,689	130,610	2.9 Å	117 Å²

^(1)^ The overall resolution was estimated by Fourier Shell Correlation of the masked half-maps after gold standard processing. ^(2)^ The B-factor of the signal decay was estimated [52] during post-processing in relion 3.1. ^(3)^ Movies were acquired at 300 kV with 20 frames per movie and a total exposure of 40 e/Å² using a Falcon III camera in integrating mode. The calibrated pixel size was 1.0635 Å [37]. The targeted under-focus was between 0.6 and 1.4 µm.

## Data Availability

Cryo-EM maps reported in this article have been deposited in the Electron Microscopy Data Bank (EMDB) with ID-codes: EMD-13726 (HBc-F97L), EMD-13728 (HBc-F97L+TX100), EMD-13731 (HBc +TX100), EMD-13734 (HBc +TX100+SLLGRM), EMD-13732 (HBc-L60V +TX100) and EMD-13733 (HBc-P5T +TX100). The molecular models were deposited in the Protein Data Bank (PDB) with ID-codes: PDB-7PZ9 (HBc-F97L), PDB-7PZI (HBc-F97L+TX100), PDB-7PZK (HBc +TX100), PDB-7PZN (HBc +TX100+SLLGRM), PDB-7PZL (HBc-L60V +TX100) and PDB-7PZM (HBc-P5T +TX100).
